# Enhanced Gait Variability Index in Neurological and Geriatric Rehabilitation: A Scoping Review

**DOI:** 10.1177/10538135261465012

**Published:** 2026-07-08

**Authors:** Mengdi Wang, Priya Karakkattil

**Affiliations:** 1 School of Physical Therapy, Texas Woman's University, Dallas, TX, USA

**Keywords:** gait variability, enhanced gait variability index, gait assessment, Parkinson's disease, aging, neurological rehabilitation

## Abstract

**Background:**

Gait variability is a sensitive marker of neuromotor function and a clinically relevant indicator of fall risk and mobility impairment in neurological and geriatric populations. The Enhanced Gait Variability Index (eGVI) has emerged as a promising composite measure that captures multidimensional gait irregularity in a single interpretable score, offering a practical advantage over single-parameter approaches. Despite growing clinical interest, the evidence base supporting its standardized implementation has not been mapped.

**Objectives:**

To characterize populations and clinical contexts where the eGVI has been applied, map assessment protocol heterogeneity, and identify methodological gaps limiting precision medicine-aligned implementation in neurological and geriatric rehabilitation.

**Methods:**

A scoping review following PRISMA-ScR guidelines was conducted using PubMed, CINAHL, Scopus, Emcare, and Web of Science. Peer-reviewed studies reporting eGVI outcomes were included. Data were extracted on participant characteristics, gait assessment protocols, and outcomes, with attention to factors influencing clinical interpretation.

**Results:**

Eighteen studies involving 1,915 participants were included, predominantly examining Parkinson's disease (39%) and older adult populations (22%). The eGVI demonstrated preliminary sensitivity to neuromotor impairment, age-related gait changes, and increased task complexity during dual-task walking; findings were most consistent in Parkinson's disease, based on limited number of studies (n = 7). Substantial heterogeneity in walkway systems, walking conditions, assistive device use, and reference populations limited comparability.

**Conclusion:**

While the eGVI shows promise as a clinical outcome measure, protocol variability and reliance on a single unvalidated normative reference limit its clinical use. Population-specific protocols and normative data consistent with a precision medicine approach, are essential for reliable adoption.

## Introduction

Gait variability, the natural stride-to-stride fluctuation present in every walking pattern (O'Sullivan et al., 2019), is a sensitive marker of neuromotor health. In healthy walking, optimal variability supports adaptability, allowing the neuromuscular system to use movement synergies to maintain and restore balance in response to environmental perturbations (Hausdorff, 2005; Ó'Reilly & Federolf, 2021). When this regulatory capacity is disrupted by aging or neurological diseases, gait variability becomes inconsistent, less automatic, and more vulnerable to external demands (Hausdorff, 2005; Leon & Woo, 2018; Mueller & Maluf, 2002). Clinically, altered gait variability is associated with increased risk of falls and reduced mobility (Hausdorff, 2005), while deviations from the optimal range in either direction may reflect underlying postural instability and motor impairment (Sidoroff et al., 2021), making precise quantification of gait variability a clinical and a research priority.

Beyond general aging-related decline, neurological conditions such as Parkinson's disease (PD), Multiple Sclerosis, and Huntington's disease precipitate a more fundamental breakdown in the natural rhythmic structure of locomotion, producing distinct gait signatures that set them apart from the healthy population (Moon et al., 2016). These signatures manifest as measurable, reproducible changes in stride-to-stride fluctuations that reflect the specific pattern of neuromotor dysregulation underlying each condition. In PD, gait variability markers can differentiate idiopathic disease from atypical Parkinsonian syndromes such as multiple system atrophy, and gait variability increases progressively with disease severity (Sidoroff et al., 2021). In Huntington's disease, stride-to-stride fluctuations correlate with disease progression and functional status (Gaßner et al., 2020). In Multiple Sclerosis, gait variability is sensitive to fatigue and cognitive load, revealing neuromotor deficits that standard walking speed measures alone fail to detect (Mofateh et al., 2017; Müller et al., 2021). These disease-specific signatures make gait variability a uniquely informative digital biomarker - a physiological signal that sensor-based systems can capture continuously, and that sensitively reflects the integrity of the underlying neurological control system (Baek et al., 2024; Coravos et al., 2019; Klucken et al., 2013; Moon et al., 2016; Pieruccini-Faria et al., 2021; Schaefer et al., 2015; Strongman et al., 2023). This sensitivity is further amplified during dual-task walking, where dividing cognitive resources leads to immediate deterioration that predicts falls more accurately than single-task performance in both older adults and in neurological populations (Mofateh et al., 2017; Yogev et al., 2005).

Accurate quantification of gait variability requires appropriate measurement tools, and the choice of system has direct implications for clinical applicability. Instrumented walkways such as Zeno and GAITRite systems detect discrete footfall events and capture spatiotemporal parameters with high validity in controlled clinical environments (Bilney et al., 2003; Vallabhajosula et al., 2019). Motion capture systems offer superior kinematic accuracy but are constrained by cost, setup complexity, and the need for specialist operators (Simon, 2004), limiting their use outside research settings. For more accessible assessment, wearable accelerometers enable continuous monitoring during natural walking conditions, capturing stride-to-stride fluctuations that laboratory snapshot assessments may miss (Del Din et al., 2016). Smartphone-based accelerometers offer a low-cost alternative (Baek et al., 2024), although they sacrifice some spatial precision (Strongman et al., 2023). The selection of an appropriate system should be guided by the clinical context, the population of interest, and the specific gait parameters under investigation. However, the clinical value of any measurement system depends on the validity and standardization of the analytical framework applied to its output.

Gait variability has traditionally relied on single-parameter metrics such as standard deviation and coefficient of variation of stride time and stride length, which provide an incomplete picture of the multidimensional nature of walking (Ellis et al., 2015; Gaßner et al., 2020; Karatsidis et al., 2025). To address this, Gouelle et al. (2013) introduced the Gait Variability Index (GVI), a composite measure derived from nine spatiotemporal parameters, including step length, stride length, step time, stride time, swing time, stance time, single support time, double support time, and walking velocity (Gouelle et al., 2013). However, redundancy within the parameter set limited its discriminative power (Gouelle et al., 2018). Gouelle et al. (2018) subsequently developed the Enhanced Gait Variability Index (eGVI), retaining only five non-redundant parameters (step length, step time, stance time, single support time, and stride velocity) and merging data from both limbs to improve clinical interpretability. Scores are normalized against a healthy normative group aged approximately 12–62 years (Gouelle et al., 2013), where 100 represents typical gait variability with a standard deviation of 10, and higher scores are associated with elevated falls risk (Gouelle et al., 2018). The eGVI offers rehabilitation clinicians a single interpretable composite score that reflects multidimensional gait irregularity, representing a meaningful advance over both single-parameter metrics and the redundancy-affected GVI. At the same time, composite indices carry inherent trade-offs. Collapsing several gait parameters into a single value may obscure which specific impairment is driving a given score, limiting interpretability at the individual-patient level. It also remains unclear whether this added complexity improves clinical decision-making relative to simpler, more transparent single-parameter metrics. These considerations make it important to examine how, and how consistently, the eGVI has actually been applied in practice.

Despite the methodological advance, the eGVI's translation into clinical rehabilitation practice remains limited, and three gaps are particularly consequential for clinical adoption. First, the clinical interpretation of eGVI scores remains uncertain, as score thresholds have not been established for specific clinical populations. Second, acquisition protocols vary widely across studies, with no standardized approach to walkway type, walking distance, or assistive-device use. Third, the eGVI relies on a single normative reference that has not been validated for the clinical populations to which it is applied. Since its introduction in 2018, the eGVI has been applied across a growing range of neurological and geriatric conditions, yet no synthesis of this literature has addressed these gaps.

This review aims to: (1) characterize the populations and clinical contexts in which the eGVI has been applied; (2) map the heterogeneity of current assessment protocols; and (3) identify methodological gaps that must be addressed to support precision medicine-aligned implementation of the gait assessment in neurological and geriatric rehabilitation.

## Methods

This scoping review followed the methodological framework for scoping reviews and is reported in accordance with the Systematic Reviews and Meta-Analyses extension for Scoping Reviews (PRISMA-ScR) checklist. The protocol was registered on Figshare (registration number:10.6084/m9.figshare.29537939).

### Eligibility Criteria

Studies were eligible for inclusion if they (1) employed the eGVI as an outcome measure, predictor variable, or in psychometric evaluation; (2) included adult aged ≥ 18 years; (3) examined populations with neurological conditions or age-related changes; and (4) were peer-reviewed articles published in English. Hierarchical exclusion criteria were applied to ensure consistent screening. Studies were excluded if they (1) were not published in English, (2) were not full-text peer-reviewed publications (e.g., conference abstracts, editorials, commentaries, or dissertations), (3) involved pediatric populations, (4) did not include neurological or older adult populations, or (5) did not report the eGVI in one of the eligible outcomes.

### Search Strategy

**Figure 1.**
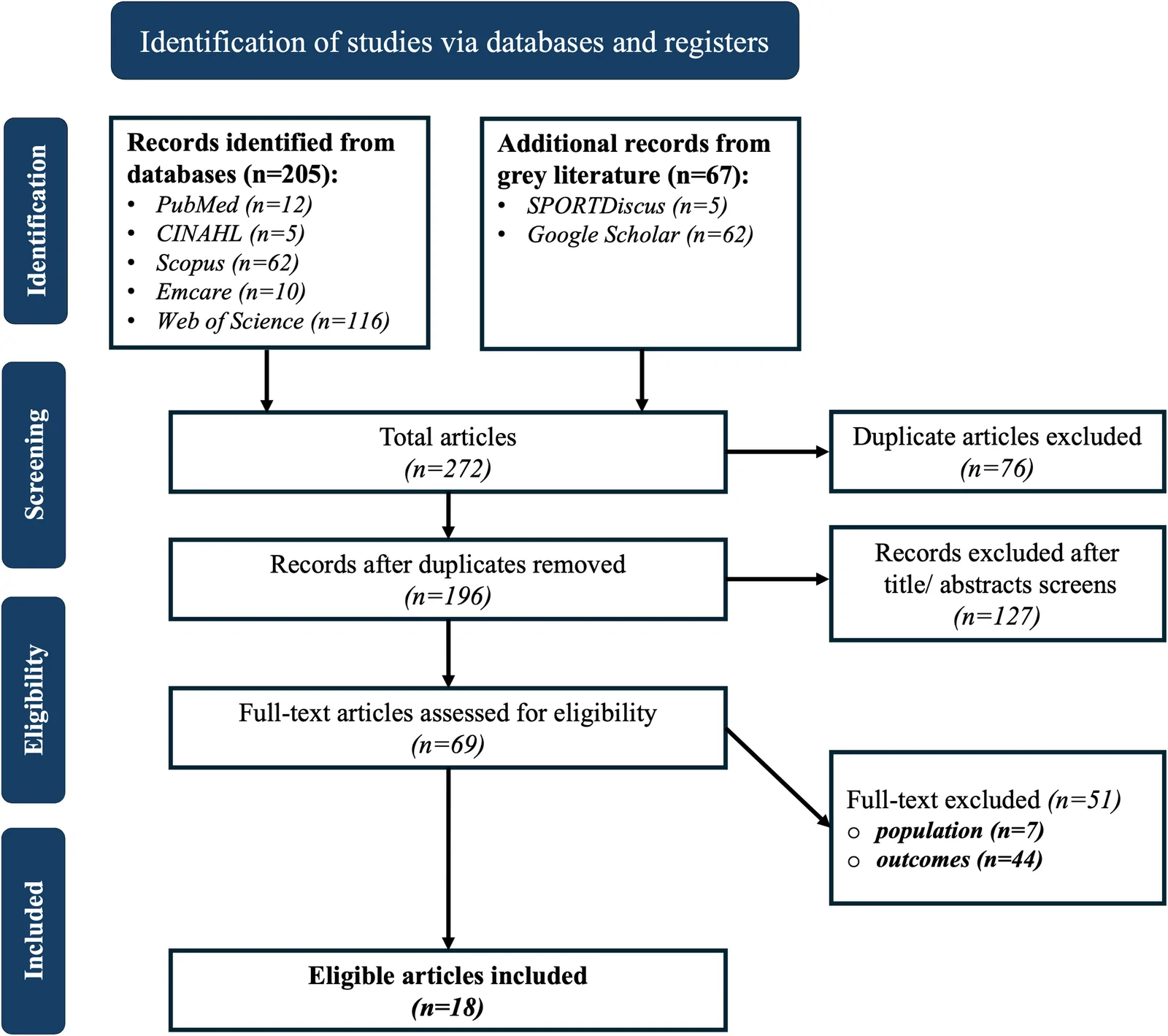
Flowchart illustrating the screening process for study selection (PRISMA-ScR).

A comprehensive literature search was conducted on July 13, 2025, and repeated on February 1, 2026, across PubMed, CINAHL, Scopus, Emcare, and Web of Science (M.W.), complemented by grey literature searches in SPORTDiscus and Google Scholar. The same search string, comprising the terms “Enhanced Gait Variability Index” OR “eGVI”, was applied across all sources, combined with population terms: “neurological”, “aging”, “seniors”, “older adults”. No date restrictions were applied, but results were limited to English-language studies involving human participants. A pilot search was undertaken to refine the strategy before full implementation. Grey literature records were screened against the same inclusion and exclusion criteria as the database records, and the full selection flow is shown in Figure 1.

### Study Selection

Search results were imported into a systematic review management platform (Rayyan Systems Inc., Doha, Qatar) for deduplication and management. Two reviewers (M.W. and P.K.) independently screened titles and abstracts against inclusion criteria. Full-text assessments were conducted for potentially eligible studies with disagreements resolved through discussion and consensus. The study selection process is summarized in the PRISMA flow diagram (see Figure 1).

### Data Analysis

Data from all eligible studies were manually extracted by one reviewer (M.W.) into a Microsoft Excel, capturing authors, publication year, study design and aims, participant and assessment characteristics, walking protocols, eGVI outcomes, and key findings, and were independently validated by a second reviewer (P.K.). Discrepancies were resolved through discussion and consensus. Owing to methodological heterogeneity, results were summarized descriptively in tables and complemented by a narrative synthesis.

## Results

Eighteen studies published between 2019 and 2026 met the eligibility criteria, collectively encompassing 1,915 participants. The initial database search identified 272 records, which were reduced to 196 studies after removing duplicates in Rayyan. Title and abstract screening reduced the number of studies eligible for full-text review to 69, of which 18 met the inclusion criteria. The remaining 51 studies were excluded, including 7 studies with the wrong population, and 44 studies investigating unrelated outcomes of which 1 study focused on the development of the eGVI methodology.

### Characteristics of Included Studies

All 18 eligible studies were published between 2019 and 2026, following the introduction of the eGVI in 2018. Included study characteristics are summarized in a chronological order (see Table 1). Blank cells in the table indicate information that was not reported in the original publications. Seventy-two percent of studies used an observational design, while the remaining implemented an interventional approach. Study locations were identified primarily from the methods sections or, when unspecified, inferred from author affiliations.

**Table 1.** Characteristics of the Included Studies.

Authors (year)	Country	Study Design	Aim (s)	Etiology	Total N	Etiology Group	Reference Group
Gilmore et al. (2019)	Canada	Cross-sectional	Examine the clinical value of backward walking assessment and compare gait responses to Levodopa during forward and backward walking.	PD	73	n = 62 (32%F) 65.29 ± 7.17 yrs	n = 11 healthy controls (36%F) 63.09 ± 8.09 yrs
Schmitt et al. (2020)	USA	Retrospective cross-sectional	Compare individual STPs and the eGVI as predictors of fall frequency in PD.	PD	273	n = 273 (31%F) 68 ± 10 yrs	
Kalsi-Ryan et al. (2020)	Canada	Prospective cross-sectional	Assess associations between mJOA gait impairment scores and objective gait parameters and characterize the severity of DCM using STPs.	DCM	166	n = 153 (46%F) 56.81 ± 10.92 yrs Mild: Mod: Sev =54%: 26%: 20%	n = 13 healthy controls 56.8 ± 6.8 yrs
Jabbar et al. (2020)	Singapore	Cross-sectional	Derive the eGVI reference values in an Asian population and evaluate its ability to distinguish between levels of physiological fall risk.	Older adults	511	n = 243 (56%F) HR:LR = 61:182 -HR = 79.78 ± 5.70 yrs -LR = 73.52 ± 5.56 yrs	n = 268 healthy controls (58%F) 43.82 ± 13.80 yrs
Martin et al. (2021)	Canada	Ambispective longitudinal cohort study	Define the natural history of nonoperatively managed DCM and evaluate the utility of quantitative clinical measures and MRI in detecting neurological deterioration.	DCM	117	n = 117 (46%F) 54.6 ± 10.6 yrs	
Berner et al. (2021)	South Africa	Cross-sectional	Investigate gait biomechanics and variability and identify a sensitive clinical screening test for early functional decline in people with HIV.	HIV	100	n = 50 (58%F) 36.6 yrs	n = 50 SNP (80%F) 31.1 yrs
Shearin et al. (2021)	USA	Quasi-experimental	Assess the impact of a community-based boxing program on STPs during normal, dual-task, and backward walking in individuals with mild to moderate PD.	PD	26	n = 26 (23%F) 68.38 yrs	
Jabbar et al. (2022)	Singapore	Cross-sectional	Examine whether the eGVI predict cognitive decline among at-risk community-dwelling adults, and whether it provides additional or distinct information from that predicted with gait speed.	Older adults	311	n = 311 (55% F) 61.46 ± 18.67 yrs	
Selvarajah et al. (2022)	Canada	Prospective cross-sectional	Examine gait and motor manifestations in adults with Dravet Syndrome (DS) and their progression with age.	DS	98	n = 17 (6 of 17 prospective) 31.81 ± 9.6 yrs	n = 81 healthy controls 62 yrs
Tiernan et al. (2022)	USA	Cross-sectional	Examine the dual-task cost of the eGVI, its component STPs, and other common gait parameters in community-dwelling older adults.	Older adults	38	n = 38 (74% F) 72.5 ± 4.8 yrs	
Zorkot et al. (2023)	Brazil	Cross-sectional (case series)	Explore STPs and COP associated with wearable ankle exoskeleton (G-Exos) use after stroke.	Stroke	3	n = 3 (67%F) 52–63 yrs	
Pretzer-Aboff et al. (2024)	USA	RCT	Identify optimal vibration therapy parameters to improve gait disturbances and assess the safety and tolerability of the intervention in PD.	PD	26	n = 26 (12%F) 69.4 ± 8.2 yrs	
Herrin et al. (2024)	USA	Cross-over	Compare community mobility outcomes during walking with and without a smart hip exoskeleton after stroke.	Stroke	9	n = 9 (100%M) 52.2 ± 11.3 yrs	
Conklin et al. (2025)	USA	Cross-sectional secondary analysis	Identify gait characteristics associated with FOG severity and distinguish individuals with and without FOG across ON- and OFF-medication states.	PD	36	n = 15 with FOG (27%F) 70.67 ± 5.80 yrs	n = 21 without FOG (33%F) 69.10 ± 6.70 yrs
Castillo et al. (2025)	USA	Cross-sectional	Examine the relationships between concussion history, concussion education, and athletic background with academic performance and single- and dual-task performance in graduate healthcare students.	Graduate students	32	n = 32 (63%F) 25 ± 2.46 yrs 34% concussion history	
Salimi et al. (2026)	Canada	RCT Secondary analysis	Examine associations between STPs and adaptations in fall prediction after mixed power training.	Older men	21	n = 12 responders (100%M) 66.5 ± 5.7 yrs	n = 9 non-responders (100%M) 67.7 ± 6.5 yrs
Peoples et al. (2026)	USA	Prospective RCT	Examine whether STN DBS improves gait variability and whether directional stimulation is superior to ring stimulation, and to assess associations between baseline eGVI and clinical outcomes.	PD	28	n = 12 elevated eGVI 61 ± 7 yrs	n = 16 normative eGVI 58 ± 8 yrs
Chiu et al. (2026)	USA	Cross-sectional	Examine effects of dual-task walking with varying task complexity on speech and gait parameters in individuals with PD and explore predictors of dual-task performance.	PD	47	n = 36 (65%F) 67.8 ± 7.5 yrs	n = 11 healthy controls (45%F) 66.7 ± 8.6 yrs;

Abbreviations: N/n = number; USA = United States of America; RCT = randomized clinical trial; M = male; F = female; eGVI = enhanced gait variability index; STPs = spatiotemporal gait parameters; COP = center of pressure; MRI = magnetic resonance imaging; PD = Parkinson's disease; FOG = freezing of gait; DBS = deep brain stimulation; STN = subthalamic nucleus; DCM = Degenerative Cervical Myelopathy; HIV = Human Immunodeficiency Virus; SNP = HIV-seronegative participants; DS = Dravet Syndrome; mJOA = modified Japanese Orthopedic Association; Mod = moderate; Sev = severe; HR = high fall risk; LR = low fall risk.

### Population

In total, 1,915 participants were analyzed in this review, aged 21 to 90 years. In studies with clinical conditions (n = 13), the mean age was 60.95 years. Among studies reporting gender (n = 15), 45% of participants in the clinical groups were female. Two studies did not report gender (Peoples et al., 2026; Selvarajah et al., 2022). A further study lacked subgroup data (Castillo et al., 2025) and was excluded from the pooled calculation of gender percentages. Diagnoses included PD (n = 7) (Chiu et al., 2026; Conklin et al., 2025; Gilmore et al., 2019; Schmitt et al., 2020; Shearin et al., 2021; Peoples et al., 2026; Pretzer-Aboff et al., 2024), degenerative cervical myelopathy (n = 2) (Kalsi-Ryan et al., 2020; Martin et al., 2021), adults with Dravet Syndrome (n = 1) (Selvarajah et al., 2022), stroke (n = 2) (Herrin et al., 2024; Zorkot et al., 2023), and Human Immunodeficiency Virus (n = 1) (Berner et al., 2021). Additionally, older adults (n = 4) (Jabbar et al., 2020, 2022; Salimi et al., 2026; Tiernan et al., 2022), and graduate healthcare students (n = 1) were also included in this review (Castillo et al., 2025). Fifty percent of 18 studies incorporated control groups with clinical populations.

### Walking Protocols

Walking protocols varied considerably across the included studies (see Table 2). All studies assessed forward walking at a self-selected speed, while 7 studies included additional conditions to examine task-dependent variability, including backward walking (n = 3) (Castillo et al., 2025; Gilmore et al., 2019; Shearin et al., 2021), dual-task walking (n = 5) (Berner et al., 2021; Castillo et al., 2025; Chiu et al., 2026; Shearin et al., 2021; Tiernan et al., 2022), or fast-paced walking (n = 3) (Berner et al., 2021; Peoples et al., 2026; Shearin et al., 2021). Participants walked barefoot in 4 studies (Berner et al., 2021; Jabbar et al., 2020, 2022; Kalsi-Ryan et al., 2020), and the use of assistive devices was restricted in 2 studies (Kalsi-Ryan et al., 2020; Schmitt et al., 2020), whereas the remaining studies did not report these specific conditions. The number of walking trials also varied, as one study conducted two trials (Pretzer-Aboff et al., 2024), 5 studies conducted three trials (Berner et al., 2021; Castillo et al., 2025; Chiu et al., 2026; Jabbar et al., 2020; 2022), and 7 studies conducted four trials. Two studies reduced the number of trials as task complexity increased (Gilmore et al., 2019; Shearin et al., 2021), such as when changing walking direction or incorporating dual-task activities, whereas 1 study used a single trial per condition (Conklin et al., 2025), and 2 studies did not specify the number of trials (Martin et al., 2021; Salimi et al., 2026). Data were primarily collected using pressure-sensitive walkways, with Zeno used in 13 studies, GAITRite in 2 studies, and both systems used in combination in 2 studies. One study used a three-dimensional portable motion-capture system (Berner et al., 2021).

**Table 2.** Gait Assessment Equipment & Walking Protocols.

Study	Equipment	Analysis System	Walkway Length	Walking Speed	Walking Direction	Task	Trial
Gilmore et al. (2019)	Zeno	PKMAS	6.1 m mat	SSV	FW & BW	ST	5x FW, 4x BW
Schmitt et al. (2020)	Zeno	PKMAS	8 m mat; 1 m accel./decel. zones	SSV (no AD)	FW	ST	4x
Kalsi-Ryan et al. (2020)	Zeno & GAITRite	PKMAS	8 m mat	SSV (barefoot, no AD)	FW	ST	4x
Jabbar et al. (2020)	GAITRite	R Studio (macro)	6 m mat; 1 m accel./decel. zones	SSV (barefoot)	FW	ST	3x
Martin et al. (2021)	Zeno & GAITRite	PKMAS					
Berner et l. (2021)	3D motion capture (myoMotion IMU)	Excel (macro)	10 m walkways; 1 m accel./decel. zones	SSV & FV (barefoot)	FW	ST & DT (count backward)	ST: 3x SSV, 3x FV DT: 3x SSV
Shearin et al. (2021)	Zeno	PKMAS	20 ft (6.1 m) mat	SSV & FV	FW & BW	ST & DT (count backward in 3 s from 100)	ST FW: 4× SSV, 4× FV ST BW: 2× SSV DT FW: 2× FV
Jabbar et al. (2022)	GAITRite	R Studio	6 m mat; 1 m accel./decel. zones	SSV (bare foot)	FW	ST	3x
Selvarajah et al. (2022)	Zeno	PKMAS	6 ft (1.83 m) mat		FW	ST	4x
Tiernan et al. (2022)	Zeno	PKMAS	6.1 m mat; 2 m accel./decel. zones	SSV	FW	ST & DT (carry a tray with a marble within square)	4x ST, 4x DT
Zorkot et al. (2023)	Zeno	PKMAS	6 m mat; 1 m accel./decel. zones	SSV	FW	ST	4x
Pretzer-Aboff et al. (2024)	Zeno		20 ft (6.1 m) mat; 24 ft walk path	SSV	FW	ST	Time-based protocol; number of passes not specified.
Herrin et al. (2024)	Zeno	PKMAS	4.8 m mat; 10 m walk path	SSV	FW	ST	4x
Conklin et al. (2025)	Zeno	PKMAS	8 m mat	SSV	FW	ST	Test both ON-Med and OFF-Med; number of passes not specified.
Castillo et al. (2025)	Zeno	PKMAS	2 ft accel./decel. zones	SSV	FW & BW	ST & DT (Auditory Stroop; Memory/Recall; Visuospatial)	ST: 3x FW, 3x BW DT: each, 3x FW, 3x BW Auditory Stroop Memory/Recall Visuospatial
Salimi et al. (2026)	Zeno	PKMAS		SSV	FW	ST	Not reported; gait analyzed during three 2-min phases of the 6MWT.
Peoples et al. (2026)	Zeno	PKMAS	5.5 m mat; 10 m walk path	SSV & FV	FW	ST	4 x SSV, 4 x FV
Chiu et al. (2026)	Zeno	PKMAS	20 ft (6.1 m) mat; 30 ft walk path with turns	SSV	FW & Figure-8	ST & DT (spontaneous monologues; serial counting)	ST: 3x FW, 3x Figure-8 DT: 3x FW, 3x Figure-8

Abbreviations: eGVI = enhanced gait variability index; PKMAS = ProtoKinetics Movement Analysis Software; accel. = acceleration; decel. = deceleration; SSV = self-selected walking speed; FV = fast walking speed; FW = walking forward; BW = walking backward; ST = single task; DT = dual task; Med = medication; 6MWT = 6-Minute Walk Test.

### Outcome Measures

The eGVI was applied for three primary analytical purposes across the 18 studies (see Table 3). Six studies utilized the eGVI as a predictor variable, examining its associations with cognitive function (Jabbar et al., 2022), falls (Jabbar et al., 2020; Schmitt et al., 2020), disease progression (Kalsi-Ryan et al., 2020; Martin et al., 2021), and concussion-related variables (Castillo et al., 2025). Five studies evaluated the psychometric or discriminative properties of the eGVI (Berner et al., 2021; Chiu et al., 2026; Gilmore et al., 2019; Shearin et al., 2021; Tiernan et al., 2022). The remaining studies measured the eGVI to evaluate responsiveness to interventions or experimental conditions, including exercises (Salimi et al., 2026; Shearin et al., 2021), medication effects (Conklin et al., 2025), exoskeleton-assisted walking (Herrin et al., 2024; Zorkot et al., 2023), vibration therapy (Pretzer-Aboff et al., 2024), and deep-brain stimulation effects (Peoples et al., 2026).

**Table 3.** eGVI Score & Findings.

Study	Etiology	eGVI (Mean ± SD)	Results	Findings
Gilmore et al. (2019)	PD	FW vs BW PD-OFF: 114.006 ± 15.361vs 138.938 ± 6.941 PD-ON: 108.069 ± 12.530 vs 137.143 ± 7.252 Control: 97.549 ± 3.459 vs 131.937 ± 7.496	The comparison of PD-ON and Control were significant in both walking tasks (*p* < 0.01, *p* < 0.05), and the comparison of PD-OFF and PD-ON was significant in forward walking (*p* < 0.01) but not in backward walking. Factor Analysis: OFF (FW): Factor 1 (Variability) – 30% variance; eGVI loading = 0.854 OFF (BW): Factor 1 (Variability) – 17.8% variance; eGVI loading = 0.626 ON (FW): Factor 1 (Variability) – 21.6% variance; eGVI loading = 0.836 ON (BW): Factor 1 (Variability) – 25.0% variance; eGVI loading = 0.900	eGVI improved in PD with levodopa in forward but not backward walking. eGVI remained elevated versus controls. Backward walking captured medication-resistant deficits.
Schmitt et al. (2020)	PD	Total eGVI = 112 ± 12 No falls = 110 ± 11 Rarely falls = 113 ± 11 Monthly falls = 123 ± 14 Weekly falls = 119 ± 12 Daily falls = 130 ± 14	eGVI predicted 15.1% of variance in fall frequency (*p* < 0.001, r = 0.389). Individual gait parameters combined predicted only 6.9% (*p* = 0.002, r = 0.264). No individual gait parameter reached significance. No falls & rarely falls groups had significantly lower eGVI than monthly and daily fallers (*p* < 0.008). No significant difference in eGVI between monthly and daily fallers.	eGVI is a better predictor of fall frequency in persons with PD than its individual spatiotemporal components. Patients who are falling more frequently have more variable gait.
Kalsi-Ryan et al. (2020)	DCM	Control = 103.36 ± 4.54 DCM = 117.54 ± 13.5 Mild = 110.9 ± 9.73 Mod = 119.14 ± 10.14 Sev = 132.94 ± 12.78	Strong negative correlation between eGVI and mJOA score (|R| = 0.551, *p* < 0.001) and mJOA LE subscore (|R| = 0.567, *p* < 0.001). Significant eGVI differences between all severity groups (mild/mod, mod/sev: *p* < 0.001, strong effect size ε^2^ = 0.36). 48.7% of mild DCM, 21.2% of moderate DCM, 0% of severe DCM had eGVI within control range. Velocity, step length, stride velocity also discriminated between groups but with weaker effect sizes.	eGVI was the most discriminative gait parameter, which facilitated objective differentiation between varying severities of DCM.
Jabbar et al. (2020)	Older adults	LR vs HR vs Control: 105.03 ± 9.18 vs 113.61 ± 10.72 vs 101.10 ± 6.70	Higher eGVI in HR vs LR and ≥65 vs <65 yrs (both *p* < 0.001); independent predictor beyond gait speed (β = 0.297, *p* < 0.001; model improvement *p* = 0.0143; R^2^ = 33.2%). AUC = 0.807; ΔAUC = 0.002 (*p* = 0.621). Moderate correlations with TUG (r = 0.554–0.679) and 5×SS (r = 0.337–0.462), all *p* < 0.01. Non-linear age–gait speed relationship: stable <60 yrs, constant >120 cm/s.	eGVI discriminated between older adults with and without high fall risk and showed moderate positive correlation with functional mobility tests in both groups. Population-specific Asian norms required for clinical application were established.
Martin et al. (2021)	DCM	Not reported.	eGVI had a sensitivity of 44% and specificity of 92% for detecting neurological deterioration in degenerative cervical myelopathy. eGVI was among the more sensitive measures compared to mJOA score (33% sensitivity), GRASSP power (0%), GRASSP sensation (10%), Berg balance scale (31%), and QuickDASH (36%). However, eGVI was less sensitive than grip strength (60%), GRASSP dexterity (60%), and gait stability ratio (50%).	eGVI has moderate sensitivity but high specificity for detecting neurological deterioration in DCM.
Berner et al. (2021)	HIV	PWH vs SNP (median) SSV: 114.66 vs 99.96 DT: 120.11 vs 113.74 In PWH, Fear Of Falling vs No Fear Of Falling SSV: 117.0 ± 12.2 vs 114.04 ± 12.0 DT: 124.0 ± 7.0 vs 119.0 ± 11.9 Fallers vs Non-fallers SSV: 112.6 ± 13.0 vs 115.6 ± 11.6 DT: 119.6 ± 12.0 vs 120.4 ± 10.9	eGVI was significantly higher in PWH than SNP during usual walking (MD = 14.7, *p* < 0.001). Dual-task eGVI was higher in PWH but not statistically significant (*p* = 0.052). 5STS moderately correlated with SSV eGVI (r = -0.5), DT eGVI (r = -0.6), self-reported mobility (r = 0.4), self-care (r = 0.5), and Fear Of Falling (*p* = 0.003). eGVI not correlated with HIV duration or antiretroviral therapy use.	PWH demonstrated slowed and variable gait under dual-task conditions. 5STS correlates with gait variability and functional impairment, offering practical screening tool for early motor decline in HIV population.
Shearin et al. (2021)	PD	Pre- vs. Post-intervention SSV: 119.79 ± 12.07 vs. 116.88 ± 11.57 BW: 137.13 ± 9.37 vs. 136.15 ± 11.25 DT: 131.97 ± 17.10 vs. 124.50 ± 12.07	eGVI score was significantly improved in dual-task walking (*p* = 0.008).	A multi-modal boxing program produced improvements in dual-task gait.
Jabbar et al. (2022)	Older adults	Total eGVI = 104.95 ± 9.47 21–30 yrs = 101.16 ± 4.74 31–40 yrs = 99.23 ± 4.87 41–50 yrs = 100.85 ± 7.18 51–60 yrs = 104.29 ± 7.12 61–65 yrs = 102.14 ± 8.96 66–70 yrs = 103.29 ± 8.06 71–75 yrs = 103.10 ± 7.50 75–80 yrs = 110.37 ± 9.94 ·≥^3^ 80 yrs = 113.80 ± 10.60	eGVI predicted visuospatial ability (β = −2.06, *p* = 0.04, R^2^ = 26.1%) and delayed memory (β = −2.82, *p* = 0.02, R^2^ = 15.3%); gait speed predicted attention (β = 2.47, *p* = 0.04, R^2^ = 30.4%). eGVI >100 group performed poorer than eGVI = 100 across all domains of except language (*p* < 0.05 for Total Scale of RBANS, Immediate Memory, Visuospatial, Attention, Delayed Memory; *p* = 0.122 for Language).	eGVI captures decline in cognitive domains (visuospatial, delayed memory) not detected by gait speed, which uniquely predicts attention. eGVI provides differential cognitive screening information beyond speed measurements for early decline detection.
Selvarajah et al. (2022)	DS	Not reported.	The eGVI score was worse in older patients with DS (r = 0.49, *p* = 0.04). Adults with DS (mean age = 31) had worse eGVI than healthy controls (mean age = 62).	Progressive gait variability worsening in adults with DS. eGVI captured age-related motor decline in DS population.
Tiernan et al. (2022)	Older adults	ST vs DT: 104.5 ± 8.4 vs 125.1 ± 10.8	A significant difference between single and dual task walking conditions for eGVI was found (*p* < 0.001). Dual task cost was highest for eGVI and stride velocity.	Older adults had increased eGVI in response to addition of motor dual task.
Zorkot et al. (2023)	Stroke	Without vs Passive vs Active vs Hybrid Subject 1: 136.18 vs 149.15 vs 139.72 vs 151.47 Subject 2: 162.94 vs 161.52 vs 165.00 vs 157.82 Subject 3: 166.48 vs 169.36 vs 172.63 vs 160.72	All participants showed eGVI ≥100	G-Exos was not able to significantly modify eGVI in a single session.
Pretzer-Aboff et al. (2024)	PD	Not reported.	Response surface analysis identified optimal vibration parameters at 275 Hz and 0.75 mm amplitude to minimize eGVI across disease stages. H & YStage II stabilized after 4 treatment sessions; stage III showed continued improvement through 8 sessions.	eGVI was minimized with consistent vibration parameters across disease severity. As severity of PD increased, it required longer optimal treatment duration.
Herrin et al. (2024)	Stroke	Baseline vs Exoskeleton: 128.875 ± 9.504 vs 126.626 ± 8.902	No significant differences in eGVI between baseline and exoskeleton conditions (*p* = 0.360). The exoskeleton reduced eGVI by 2.2 points on average. Two subjects (S02 and S05) improved eGVI by over one standard deviation (10.5% and 10.2%, respectively). No other participants had changes exceeding one standard deviation.	eGVI was not sensitive to hip exoskeleton assistance in stroke survivors during indoor walking.
Conklin et al. (2025)	PD	Not reported.	eGVI was included in ridge regression models for both ON-medication (Lambda = 7.343) and OFF-medication (Lambda = 0.316) states but was not retained in final models predicting FOG severity. However, Lasso regression (Lambda = 0.100) identified ON-state eGVI as the strongest of three variables for FOG detection. In logistic regression, ON-medication eGVI showed beta = 0.115, OR = 1.12 (95% CI: 1.01, 1.29), *p* = 0.057, with the model achieving AUC = 0.881, sensitivity = 0.81, specificity = 0.81, and 88% overall accuracy in distinguishing freezers from non-freezers.	eGVI effectively detected FOG presence but did not predict FOG severity, with ON-medication eGVI being more informative than OFF-medication eGVI for screening purposes. As a composite measure, eGVI serves as a practical single-metric screening tool, with elevated values during unfrozen walking potentially reflecting subclinical FOG pathology.
Castillo et al. (2025)	Graduate students	Not reported.	The eGVI was not a significant predictor for concussion history, concussion education, or years of contact sports (all *p* > 0.05).	eGVI has limited utility for detecting cognitive-motor deficits related to concussion or athletic backgrounds in graduate healthcare students performing dual-task activities.
Salimi et al. (2026)	Inactive older men	At baseline 6MWT, Responders vs Non-responders Start phases: 94.4 ± 3.8 vs 94.6 ± 3.1 Middle phase: 94.2 ± 3.4 vs 95.4 ± 4.1 End: 96.5 ± 2.9 vs 91.3 ± 5.8 Total: 95 ± 3.1 vs 93.7 ± 2.8	eGVI showed no significant changes following 12 weeks of MPT across any 6MWT phase (all *p* > 0.05). No correlations were found between eGVI delta changes and muscle strength improvements (*p* > 0.05, Bonferroni-adjusted). In subgroup analysis, baseline eGVI differed only at end phase between responders and non-responders (*p* = 0.031).	eGVI demonstrated limited utility as a marker of training response in inactive older males, contrasting with other gait parameters that improved post MPT. Baseline eGVI differences at end phase suggest potential fatigue-related variability rather than training adaptability.
Peoples et al. (2026)	PD	Elevated variability group vs Normative group for SSV Baseline-OFF: 137.9 ± 16.6 vs 101.6 ± 2.8 Baseline-ON: 115.6 ± 13.5 vs 102.2 ± 5.7 Ring DBS: 116.8 ± 22.8 vs 105.4 ± 5.4 Directional DBS: 113.3 ± 14.9 vs 106.8 ± 12.1 Elevated variability group vs Normative group for FV Baseline-OFF: 130.3 ± 24.8 vs 104.65 ± 7.92 Baseline-ON: 112.5 ± 14.3 vs 102.33 ± 6.25 Ring DBS: 117.15 ± 23.59 vs 105.31 ± 9.09 Directional DBS: 116.12 ± 19.71 vs 106.09 ± 9.86	Significant group-by-visit interactions were observed for eGVI at both comfortable (F(3,75) = 12.19, *p* < 0.001, η^2^*p* = 0.33) and maximum (F(3,72) = 3.78, *p* = 0.014, η^2^*p* = 0.14) walking speed conditions. The elevated variability group improved from baseline-OFF to all other conditions (all *p* < 0.05), while the normative group remained stable. No significant differences in eGVI were found between ring and directional DBS modes at comfortable (*p* = 0.91) or maximal (*p* = 0.97) walking speeds. Baseline-OFF eGVI strongly correlated with TUG (r = 0.75, R^2^ = 0.56), PIGD (r = 0.74, R^2^ = 0.55), FOG (r = 0.60, R^2^ = 0.36), and ABC (r = -0.55, R^2^ = 0.30), all *p* < 0.001.	Unilateral STN DBS improved eGVI in PD patients with elevated baseline variability but not normative variability. eGVI stratified DBS treatment response based on baseline variability and demonstrated strong correlations with established clinical mobility measures and patient-reported outcomes.
Chiu et al. (2026)	PD	PD vs Control Simple gait: ST: 108.86 ± 12.87 vs 107.96 ± 14.86 DT: 109.44 ± 13.20 vs 106.56 ± 10.46 Complex gait: ST: 155.49 ± 2.92 vs 152.71 ± 5.97 DT: 156.01 ± 4.60 vs 153.46 ± 4.34	No significant group differences in eGVI (*p* = 0.2) or task effects (single vs dual, Beta = −0.37, *p* = 0.7). Complexity significantly affected eGVI (Beta = 49, *p* < 0.001), with complex gait showing substantially worse values. Both groups demonstrated minimal dual-task cost on eGVI, with dual task effects near zero. modified attention allocation index calculations showed positive values across all comparisons. No participant characteristics significantly predicted eGVI dual-task effects in regression models.	eGVI was sensitive to gait complexity but showed minimal responsiveness to dual-task speech demands in PD.

Abbreviations: eGVI = enhanced gait variability index; MD = mean difference; FW = walking forward; BW = walking backward; ST = single task; DT = dual task; SSV = self-selected walking speed; FV = fast walking speed; PD = Parkinson's disease; FOG = freezing of gait; DBS = deep brain stimulation; STN = subthalamic nucleus; DCM = Degenerative Cervical Myelopathy; HIV = Human Immunodeficiency Virus; PWH = people live with HIV; SNP = HIV-seronegative participants; DS = Dravet Syndrome; mJOA = modified Japanese Orthopedic Association; LE = lower extremity; Mod = moderate; Sev = severe; HR = high fall risk; LR = low fall risk; TUG = Time to Go test; 5XSS/5STS = 5-Times Sit-to-Stand test; BSS = Berg Balance Scale; 6MWT = 6-Minute Walk Test; MPT = mixed power training; PIGD = Postural Instability and Gait Disorder; ABC = Activities-specific Balance Confidence.

## Discussion

This scoping review represents the first systematic synthesis of research exploring the eGVI as an outcome in neurological populations and older adults since its introduction in 2018.

### Population

All 18 studies evaluated eGVI scores against the normative reference established by Gouelle et al., in which a score of 100 represents typical gait variability in a healthy population with a standard deviation of 10. The consistent use of this single reference across studies reflects a shared assumption providing a universal benchmark for gait variability. No study examined whether this reference is appropriate for the populations under investigation. Evidence from one study conducted in an Asian population (Jabbar et al., 2022) reported eGVI scores that differed from those observed in Western cohorts, raising questions about the assumption of generalizability across ethnicities, despite the single study findings requiring confirmation. Those populations differ substantially from the healthy reference group, in terms of age, ethnicity, diagnosis, and functional capacity. None of the reviewed studies established reference values for younger versus older adults, different gait speeds, walking directions, task challenges, or gender-specific thresholds. Among studies reporting gender, 45% of participants in clinical groups were female. No study conducted gender-stratified analyses, despite established gender differences in gait biomechanics and changes with aging (Johansson et al., 2016). Fall risk is disproportionately high in female rehabilitation populations (Johansson et al., 2016), and whether eGVI thresholds for elevated fall risk differ between genders remains an unanswered clinical question.

Study populations were substantially older than the healthy reference group used for eGVI calculation, with a mean age of 60.95 years in clinical studies and an age range spanning 21 to 90 years across all included studies. This age discrepancy aligns with observed eGVI elevations, as gait variability naturally increases with age-related declines in sensorimotor integration and executive control (Nóbrega-Sousa et al., 2020). This consistent pattern across the included studies suggests that eGVI detects age-related neuromotor decline. However, none of the reviewed studies established age-stratified reference values. The degree to which eGVI elevations reflect true pathology versus expected aging therefore remains unresolved. This gap is particularly consequential in older adult rehabilitation, where distinguishing pathological from age related physiological gait change directly informs intervention decisions. Only four studies incorporate younger participants, which further limits the generalizability of current eGVI evidence to younger populations.

Among the neurological conditions represented in this review, PD was the predominant focus. The eGVI in these studies consistently showed sensitivity to PD-related gait impairments, including reduced speed, asymmetry, and disrupted rhythm (Zhang et al., 2022). This supports its potential as a monitoring tool in Parkinsonian rehabilitation. While gait variability impairments are well-documented in conditions such as stroke (Balasubramanian et al., 2009), Multiple Sclerosis (Socie et al., 2013), Huntington's disease (Gaßner et al., 2020), cerebellar ataxia (Serrao et al., 2012), and peripheral neuropathy (Dingwell & Cavanagh, 2001), the eGVI has not been systematically applied across these populations. Each condition damages distinct features of gait rhythm, symmetry, and timing that differ from the Parkinsonian pattern. Whether the eGVI captures disease-specific gait signatures or reflects a more general measure of neuromotor dysregulation cannot be determined from the current literature. Rehabilitation units routinely manage patients with more complex and/or mixed neurological diagnoses, and this gap means that the eGVI cannot yet be applied with equal confidence across all clinical populations.

### Standardized Protocol

Inconsistent acquisition protocols represent a fundamental barrier to the clinical implementation of the eGVI. All 18 studies assessed forward walking at self-selected speed in controlled laboratory environments, approximating overground walking while capturing natural gait variability. However, laboratory investigations do not fully reflect daily-life walking, with notable differences between these environments existing especially in the elderly and neurologic population (Hillel et al., 2019; Rojer et al., 2021).

Only 3 studies examined backward walking in this review, yet all reported substantially elevated eGVI relative to forward walking. This consistent finding suggests that backward walking may reveal neuromotor deficits not captured during forward walking alone, consistent with prior evidence that backward walking spatiotemporal measures decline more precipitously with age than forward walking measures (Fritz et al., 2013). Backward walking requires increased motor cortex activation and greater recruitment of sensorimotor pathways compared to forward walking (Kurz et al., 2012; Sasaki et al., 2024), which likely explains its greater sensitivity to neuromotor impairment. Despite this consistency, backward walking is absent from most eGVI protocols, representing a critical gap in current assessment practice that can lead to the systematic underestimation of the true level of gait variability. A similar gap exists for faster walking speeds. Fast walking challenges stability through rapid weight transfers and increased propulsive forces (Browne & Franz, 2017). Only 3 studies examined how gait speed variations affect eGVI scores (Berner et al., 2021; Peoples et al., 2026; Shearin et al., 2021), and their findings could not be directly compared due to differing speed increments and conditions.

Five studies in this review used dual-task paradigms that produced contrasting findings. The eGVI increased under motor dual-task conditions (Tiernan et al., 2022), consistent with the established principle that competing motor demands stress gait automaticity. Conversely, the eGVI showed near-zero responsiveness to cognitive-verbal dual-task demands (Chiu et al., 2026), suggesting that adding a verbal task does not reliably challenge the motor control processes the eGVI captures. However, gait complexity rather than cognitive load produced large and significant eGVI increases in both PD and control groups in the same study (Chiu et al., 2026). The eGVI may be more sensitive to the motor complexity of the walking task itself than to concurrent cognitive demands, a distinction with important implications for protocol design. Because these observations derive largely from a single study, they should be regarded as hypothesis-generating and in need of replication. This inconsistency, combined with limited overall application, represents a significant gap, as walking rarely occurs as an isolated motor activity in daily life. Cognitive load increases gait variability during indoor walking, whereas environmental load increases it during outdoor walking (Ho et al., 2019; Montero-Odasso et al., 2012), highlighting the distinct contributions of different task demands to gait control. Dual-task conditions are particularly valuable since they reveal gait deficits that remain concealed during single-task walking (Zukowski et al., 2021). Clinically, dual-task gait deterioration predicts falls more accurately than single-task performance in older adults (Beauchet et al., 2009; Muir-Hunter & Wittwer, 2016), and insufficient dual-task assessment risks underestimating real-world functional impairment in rehabilitation settings.

Only 4 studies standardized footwear and assistive device use. The remaining studies either did not standardize these conditions or did not report them. Barefoot walking increases joint motion, reduces ground reaction force, and lowers plantar pressure compared to shoe conditions (Franklin et al., 2015), whereas footwear or assistive devices can alter cadence and shorten stride length (Bryant et al., 2012; Franklin et al., 2015). Unstandardized footwear may introduce variability that confounds the eGVI score itself. Two studies on individuals with stroke incorporated exoskeleton-assisted walking (Herrin et al., 2024; Zorkot et al., 2023). The eGVI remained substantially elevated across all exoskeleton conditions in both studies, with minimal and non-significant reductions observed. This suggests that eGVI elevations during exoskeleton-assisted walking may reflect persistent neurological impairment rather than a failure of the device. Rehabilitation clinicians using assistive technology as an intervention should interpret elevated eGVI scores in this context with caution.

The number of walking passes used to calculate eGVI varied across studies, ranging from a single walk to multiple repeated passes. The original GVI protocol employed multiple passes (Gouelle et al., 2018), yet no consensus exists on the minimum number required for stable variability estimates. No minimum stride count was specified for clinical populations. Research in PD has established that a minimum of 40 consecutive steps is required to achieve a stable variability estimate (Rennie et al., 2017, 2018). No equivalent guideline exists for other neurological conditions. An eGVI derived from insufficient stride counts may reflect measurement noise rather than one's true gait variability. The absence of a universal minimum stride count standard is one of the most practically addressable barriers to reliable eGVI use in clinical and research practice.

### Analytical Utility

The eGVI was applied as a predictor of clinical outcomes, a psychometric measure, and an intervention outcome measure across the included studies. These applications indicate that the eGVI can capture valuable variations in gait control among neurological and aging populations, consistent with evidence that variability-based gait indices are sensitive markers of neuromotor impairment (Zhang et al., 2022). However, this diversity of analytical purpose, combined with the protocol heterogeneity described above, limits synthesis. A measure used to predict falls in one study and evaluate medication effects in another will yield meaningful individual findings, but without standardized acquisition conditions, the cumulative evidence base remains fragmented.

### Clinical Implications

Several practical considerations follow from this review, though all remain provisional given the limited and heterogeneous evidence. We recommend interpreting absolute eGVI scores cautiously and tracking within-patient change under a consistent protocol rather than relying on normative comparison. Acquisition conditions, including footwear and assistive-device use, walking direction and speed, and the number of walking passes, need to be ideally standardized before eGVI scores are compared. The eGVI appears to be most informative in PD, where the available evidence is concentrated, and may warrant more cautious interpretation in other neurological conditions where evidence remains sparse. Overall, the eGVI is a promising measure to inform clinical practice, but does not yet appear ready for use as a standalone, normatively referenced clinical outcome measure.

### Limitations

The review has several limitations. First, the evidence base is small. Only 18 studies met the inclusion criteria, and they were unevenly distributed across populations, with PD overrepresented. This imbalance limits the generalizability of our findings to other neurological and geriatric groups. Second, substantial heterogeneity in study designs, gait assessment protocols, instrumentation, outcomes, and data processing restricted our ability to draw direct comparisons or perform quantitative synthesis. Third, most included studies were conducted in controlled laboratory settings, so the extent to which eGVI findings reflect functional mobility under daily-life conditions remains unclear. In addition, the search strategy targeted adults with neurological conditions and age-related gait changes, therefore results cannot be generalized to pediatric populations, and only peer-reviewed, English-language studies were included, which may have introduced language and publication bias. Consistent with scoping review methodology, a formal critical appraisal of the included studies was not conducted, as the aim of this review was to assess the breadth of eGVI applications. A transparent screening process following PRISMA-ScR guidelines was employed to enhance reproducibility.

## Conclusions

The eGVI is a clinically relevant composite measure of gait variability that has shown preliminary sensitivity in detecting gait variability in neurological and geriatric rehabilitation populations, with the most consistent findings reported in PD. However, two critical gaps limit its clinical implementation. Protocol heterogeneity across studies undermines comparability, and the universal application of a single normative reference without validation for clinical populations raises concerns about score interpretability. Standardized acquisition protocols and population-specific normative databases are recommended before the eGVI can be adopted with confidence as a clinical outcome measure. Longitudinal evidence of responsiveness to rehabilitation-induced changes is also required to support its use as a monitoring tool. These findings underscore the need for a precision medicine approach to gait variability assessment, in which population-specific protocols and reference values replace uniform benchmarks to better reflect individualized rehabilitation.
